# Liver Expression of Sulphotransferase 2A1 Enzyme Is Impaired in Patients with Primary Sclerosing Cholangitis: Lack of the Response to Enhanced Expression of PXR

**DOI:** 10.1155/2015/571353

**Published:** 2015-10-04

**Authors:** Ewa Wunsch, Marta Klak, Urszula Wasik, Malgorzata Milkiewicz, Malgorzata Blatkiewicz, Elzbieta Urasinska, Olivier Barbier, Dariusz Bielicki, Dimitrios P. Bogdanos, Elwyn Elias, Piotr Milkiewicz

**Affiliations:** ^1^Department of Clinical and Molecular Biochemistry, Pomeranian Medical University, 70-111 Szczecin, Poland; ^2^Department of Medical Biology, Pomeranian Medical University, 70-111 Szczecin, Poland; ^3^Department of Pathology, Pomeranian Medical University, 71-252 Szczecin, Poland; ^4^Laboratory of Molecular Pharmacology, CHU Québec and Faculty of Pharmacy, Laval University, Québec, QC, Canada; ^5^Department of Gastroenterology, Pomeranian Medical University, 71-252 Szczecin, Poland; ^6^Liver Sciences, Division of Liver Transplantation and Mucosal Biology, King's College London School of Medicine, Denmark Hill Campus, London SE5 9RS, UK; ^7^Faculty of Medicine, School of Health Sciences, University of Thessaly, Larissa 41110, Greece; ^8^Liver and Hepatobiliary Unit, Queen Elizabeth Hospital, University of Birmingham, Birmingham B15 2TT, UK; ^9^Liver and Internal Medicine Unit, Department of General, Transplant and Liver Surgery, Medical University of Warsaw, 02-097 Warsaw, Poland

## Abstract

*Background/Aim*. Sulphotransferase 2A1 (SULT2A1) exerts hepatoprotective effects. Transcription of *SULT2A1* gene is induced by pregnane-X-receptor (PXR) and can be repressed by miR-378a-5p. We studied the PXR/SULT2A1 axis in chronic cholestatic conditions: primary sclerosing cholangitis (PSC) and primary biliary cirrhosis (PBC).* Materials/Methods*. Western-blot/PCRs for SULT2A1/PXR were performed in PSC (*n* = 11), PBC (*n* = 19), and control liver tissues (*n* = 19). *PXR* and *SULT2A1* mRNA was analyzed in intestinal tissues from 22 PSC patients. Genomic DNA was isolated from blood of PSC patients (*n* = 120) and an equal number of healthy volunteers. Liver miRNA expression was evaluated using Affymetrix-Gene-Chip miRNA4.0.* Results*. Increased PXR protein was observed in both PSC and PBC compared to controls and was accompanied by a significant increase of SULT2A1 in PBC but not in PSC. Decreased expression of *SULT2A1* mRNA was also seen in ileum of patients with PSC. Unlike PBC, miRNA analysis in PSC has shown a substantial increase in liver miR-378a-5p.* Conclusions. *PSC is characterized by disease-specific impairment of SULT2A1 expression following PXR activation, a phenomenon which is not noted in PBC, and may account for the impaired hepatoprotection in PSC. miRNA analysis suggests that SULT2A1 expression in PSC may be regulated by miR-378a-5p, connoting its pathogenic role.

## 1. Introduction

Primary sclerosing cholangitis (PSC) and primary biliary cirrhosis (PBC) are both immune-mediated chronic cholestatic liver conditions [1]. PSC, which is frequently seen in association with inflammatory bowel disease [2], is a chronic biliary disease which may affect both the intra- and extrahepatic biliary tree while in PBC the main damage is noted in the small and medium size intrahepatic bile ducts. Many patients affected with PSC develop progressive biliary strictures, leading to recurrent cholangitis. Both PSC and PBC lead to hepatic and systemic accumulation of toxic biliary compounds, resulting in progressive liver damage [3]. In response, several defense mechanisms are induced to prevent from liver injury. These comprise equilibrium changes in hepatobiliary transporters, downregulation of uptake systems, and induction of enzymes catalyzing detoxification processes [4–8]. The end readout of the complex interplay of these systems is prevention of and compensation for the deleterious accumulation of toxic bile acids. At the transcriptional level, the pivotal “fine-tuning” role for the homeostasis of these adaptive mechanisms is mainly played by members of the nuclear receptor (NR) family.

The pregnane X receptor (PXR; NR1I2) is a ligand-activated member of the nuclear receptor superfamily of transcription factors, which is highly expressed in human liver and gastrointestinal tract. It serves as a xenobiotic sensor which induces phase I (catalyzing hydroxylation) and phase II (catalyzing glucuronidation and sulfation) metabolism of many endogenous and exogenous compounds including bile acids (BA).

The primary bile acids in man are cholic and chenodeoxycholic acid. They are metabolized by enteric bacteria to produce the secondary bile acids deoxycholic and lithocholic, respectively. Lithocholic acid (LCA) is extremely lipophilic, rapidly partitions into membranes, and has a high potential for toxicity. Hydroxylation and sulphation of LCA greatly reduce its intestinal reabsorption, thus minimising its enterohepatic circulation and promoting its excretion in faeces. In contrast to the other bile acids which act as FXR ligands, LCA is a preferred ligand of PXR. Sonoda et al. have shown that LCA in micromolar amounts is a powerful inducer of the sulphotransferase (SULT2A1) responsible for its sulphation only in the presence of the RXR:PXR heterodimer [9]. Sulphation, a phase 2 activity, converts LCA to a less toxic and more water-soluble form which is readily excreted in faeces.

We had previously reported low levels of lithocholic acid sulphation in chronic cholestatic liver disease but could not distinguish between a causal and consequential linkage [10]. We postulated that failure of the liver's coordinated defense against lithocholic acid toxicity could be critically involved in the pathogenesis of PSC [11]. Recently, we reported that concentrations of plasma lithocholic acid sulphate were significantly reduced in PSC in comparison to patients with PBC and normal controls [12]. We postulated that an increased expression of SULT 2A1, in response to PXR activation, would be anticipated to occur as a hepatoprotective response to injurious cholestasis in conditions such as PSC and possibly PBC. To test our hypothesis, we analyzed the levels of PXR and its target gene SULT2A1 in PSC and PBC patients with comparable degrees of clinical cholestasis and sought out for potentially significant differences amongst PSC and PBC in this regard.

## 2. Materials and Methods

### 2.1. Patients Characteristic and Tissue Specimens

Liver tissue specimens were collected from explanted livers of patients with PSC (*n* = 11) and PBC (*n* = 19) who underwent liver transplantation. Control liver tissues (*n* = 19) were obtained from large margin liver resections of colorectal metastases with no microscopic changes of liver disease identified by a pathologist.  Table 1 summarizes clinical and laboratory features of patients included in the analysis of liver expression of PXR and SULT2A1.

Intestinal tissues were obtained from a group of 22 patients with PSC who underwent their routine colonoscopies. Eleven patients (8 males, 3 females; mean age 35 ± 17) had macroscopic features of ulcerative colitis (UC), called PSC + UC group, and 11 (9 males, 2 females; mean age 30 ± 9) had never been diagnosed with inflammatory bowel disease (called PSC group). All patients with PSC were treated with ursodeoxycholic acid with an average dose of 15 mg/kg. b.w. and patients with UC additionally received 5ASA (2-3 g/daily). Specimens were collected from ileum and ascending and sigmoid colon. For this part of the study, the control group comprised 14 (8 males, 6 females; mean age 50 ± 16) subjects who underwent their colonoscopies for various indications and who were found to have no macroscopic changes in their colons. Three tissue samples were obtained from each examined part of intestine. Then, biopsies were processed for future analyses, that is, either (i) stored in RNAlater for analysis of mRNA expression (AM7021; Applied Biosystems, Carlsbad, CA, USA), (ii) fixed in neutral-buffered formalin for histological assessment, or (iii) immediately frozen in liquid nitrogen for proteomic analyses. Histology was assessed by a pathologist (EU) who was blinded to clinical diagnoses of analyzed patients, according to histological grading scale introduced by Geboes et al. [13]. Briefly, according to this score, 6 histological features are assessed; these include (i) architectural changes; (ii) chronic inflammatory infiltrate; (iii) lamina propria neutrophils and eosinophils; (iv) neutrophils in epithelium; (v) crypt destruction; (vi) erosion or ulceration.

An informed consent was obtained from each patient participating in this study. The research protocol was approved by the Ethics Committee of Pomeranian Medical University and conformed to the ethical guidelines of the 1975 Declaration of Helsinki.

### 2.2. RNA Extraction and Quantification of Gene Expression

Total RNA were isolated using the RNeasy Mini kit (Qiagen, Valencia, USA), according to the manufacturer's protocol. cDNA synthesis was carried out using Superscript II RT kit (Invitrogen, Carlsbad, CA, USA) according to the protocol previously described [14] and stored at −20°C. The expression of specific target genes was measured by quantitative real-time PCR using commercially available Gene Expression Assays and 7500 Fast Real-Time PCR System (Applied Biosystems). The following assays were used in the study: PXR (Hs01114267_m1); SULT2A1 (Hs00234219_m1); and control human GAPDH (Hs99999905_m1). A 20 *μ*L reaction mixture contained 10 *μ*L of TaqMan Gene Expression PCR Master Mix (Applied Biosystems, Foster City, CA, USA), 2 *μ*L diluted cDNA template, and 1 *μ*L of the probe/primer assay mix. The fluorescence data were analyzed with 7500 Software v2.0.2. (Applied Biosystems, Carlsbad, CA, USA). The expression of target genes was calculated using the ΔΔCt method of relative quantification.

### 2.3. Protein Expression Analysis

Proteins from frozen liver and intestine tissue were extracted through homogenization in an ice-cold RIPA buffer (50 mM Tris-HCl pH = 8, 150 mM NaCl, 1% NP-40, 0,5% NaDOC, 0,1% SDS, 1 mM EDTA, 100 mM PMSF, 100 mM NaF) containing protease inhibitor cocktail and PhosSTOP (Roche Diagnostics GmbH, Mannheim, Germany). Protein quantification was made using the bicinchoninic acid assay (Micro BCA Protein Assay Kit; Thermo Scientific, Waltham, MA, USA). Forty *μ*g of protein extracts from each liver sample was electrophoresed in SDS polyacrylamide gels and subsequently blotted into PVDF membranes (Thermo Scientific, Rockford, IL, USA) under semidry transfer conditions. Membranes were blocked overnight at 4°C with TBST containing 5% (w/v) milk (Merck) and then probed using the following primary antibodies: PXR (sc-48403; Santa Cruz, 1 : 500), SULT2A1 (sc-8002 Santa Cruz, 1 : 200), and anti-*α*/*β*-tubulin (2148, Cell Signaling, 1 : 1000). For the detection of antigen-antibody complexes, peroxidase conjugated anti-rabbit secondary antibody (NA9340V, Amersham, GE Healthcare, UK; 1 : 5000 dilution) or anti-mouse secondary antibody (NA9310V, Amersham; 1 : 5000 dilution) was used. Protein expression was detected using an enhanced chemiluminescence detection system (Chemiluminescent HRP Substrate, Millipore, Billerica, MA, USA). Bands were visualized and quantified using MicroChemi 2.0 System and GelQuant software (Israel).

### 2.4. *SULT2A1* Genotyping and Promoter Sequencing

**Figure 1:**
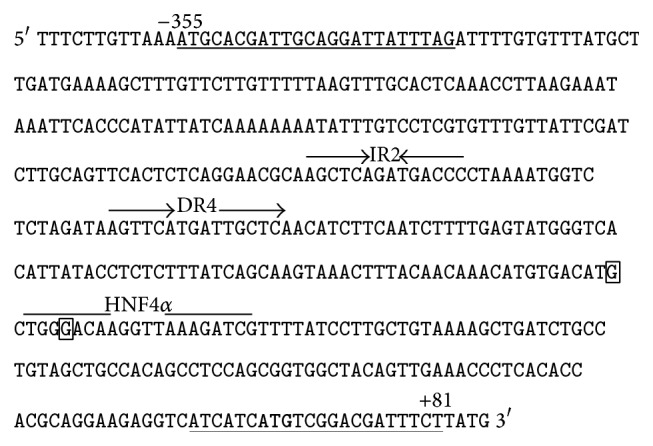
The DNA sequence within* SULT2A1* promoter region (−367 to +85). The positions of the studied SNPs, that is, rs11569683, rs112433193,  are marked in squares. The PXR binding sites are represented by IR2 and DR4 motif. The HNF4 alpha binding site is marked. The location of primers used in the sequencing of HNF4alpha and PXR binding sites is underlined.

Two SNPs (rs11569683 [A/G] and rs112433193 [C/G]), located near/within the PXR binding site within promoter region of* SULT2A1 *gene, were analyzed in genomic DNA isolated from peripheral blood mononuclear cells of 151 PSC patients (109 males, 42 females; mean age 32 ± 13), (DNeasy Blood & Tissue Kit, Qiagen). PCR reactions contained 20 ng DNA, 900 nM of each primer, 12.5 *μ*L of* TaqMan* Universal Master Mix, and 200 nM of VIC-labelled and FAM-labelled probes in 25 *μ*L-reactions. Amplification conditions were as follows: 95°C for 10 min, 40 cycles of 92°C for 15 s, and 60°C for 1 min. Oligonucleotide primers and TaqMan probes for the* SULT2A1* polymorphisms were designed and synthesized by Applied Biosystems. The fluorescence data were analyzed with allelic discrimination 7500 Software v.2.0.2. Additionally, for the purpose of detecting a DNA sequence of* SULT2A1* promoter region within the PXR binding sites represented by IR2 and DR4 motifs, the PCR reaction was performed in samples of genomic DNA of 151 PSCs patients with the use of the following* primers: Fw *5-GCACGATTGCAGGATTATTTAG-3′; Rv 5′AGAAATCGTCCGACATGATGAT-3′. The amplified DNA (436 pb) was purified with EXTRACTME* DNA GEL–OUT Kit (DNA, Gdansk, Poland)* followed by sequencing in the Laboratory of* DNA* Sequencing and Oligonucleotide Synthesis (Oligo.pl; Institute of Biochemistry and Biophysics, Polish Academy of Sciences). The location of primers used in the sequencing and PXR binding site within the promoter region of* SULT2A1* gene is showed in Figure 1.

### 2.5. MicroRNA Assay

Total RNA from liver samples explanted from patients diagnosed with PSC (*n* = 4), PBC (*n* = 4) and aged and gender-matched control donors for each disease (*n* = 4 per experimental group) was isolated with the use of miRNeasy Mini Kit (Qiagen). Microarray analysis comprised Affymetrix GeneChip miRNA 4.0 arrays and was performed by Microarray Core Boston University (http://www.bumc.bu.edu/microarray/).

### 2.6. Statistics

Data were evaluated as mean ± standard error (SE) for continuous variables. Data were analyzed using Stat-View-5 Software (SAS Institute, Cary, NC, US) and included Fisher's exact and ANOVA analysis. Correlations were assessed by parametric tests (Pearson Correlation test). A* p* value < 0.05 was considered statistically significant.

## 3. Results

### 3.1. Different Expression Patterns of* PXR* and* SULT2A1* in Patients with* PSC* and* PBC*

**Figure 2:**
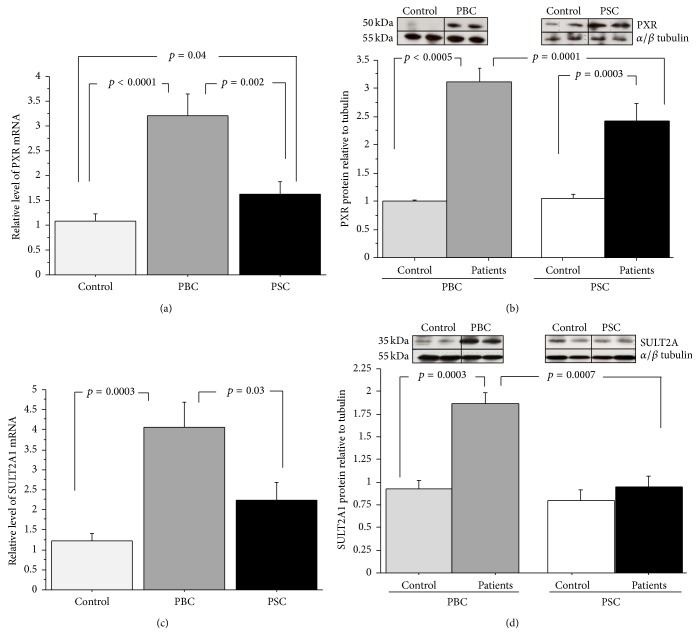
Liver expression of PXR and SULT2A1 in liver tissue from patients with cirrhotic PSC and PBC. (a) PXR mRNA, (b) PXR protein, (c) SULT2A1 mRNA, and (d) SULT2A1 protein. Levels of gene expression presented as fold-change relative to control were normalized with glyceraldehydes 3-phosphate dehydrogenase (GAPDH). Bars indicate the mean ± SEM. Changes in protein levels were determined by densitometry analyses after normalization to *α*/*β* tubulin as a control for loading. Bars indicate the mean ± SEM.

In cirrhotic liver tissues expression of* PXR* mRNA was considerably enhanced in both PSC (1.6-fold,* p* = 0,04) and PBC (3-fold increase,* p* < 0.0001 versus controls; Figure 2(a)). Expression of* PXR* mRNA did not correlate with biochemical features of cholestasis (data not shown). Similarly, protein level of PXR was significantly augmented in PSC and PBC compared to control tissues (2.7 ± 0.3 versus 1.2 ± 0.2,* p* = 0.0003 and 3.1 ± 0.5 versus 1.1 ± 0.1,* p* = 0.0005 resp.; Figure 2(b)). The increase in PXR mRNA and protein levels was significantly less pronounced in PSC than PBC patients (*p* = 0.002 and* p* = 0.0001 versus PBC, for PXR mRNA and protein levels, resp.; Figures 2(a) and 2(b)). In PBC, the enhanced PXR expression was accompanied by the increased expression of* SULT2A1* mRNA (4-fold,* p* = 0.0003 versus controls; Figure 2(c)) and SULT2A1 protein level (1.9-fold,* p* = 0.0003 versus controls; Figure 2(d)). Such changes were not observed in livers from patients with PSC (Figures 2(c) and 2(d)).

### 3.2. Expression of* SULT2A1* mRNA Is Suppressed in Small Intestine of Patients with PSC

**Figure 3:**
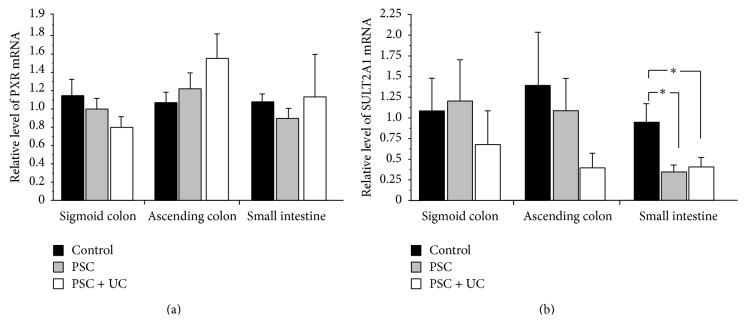
Gene expression of (a) PXR and (b) SULT2A1 mRNA in colon and small intestinal tissues of PSC patients without (PSC) or with ulcerative colitis (PSC + UC). Levels of gene expression presented as fold-change relative to control were normalized with glyceraldehydes 3-phosphate dehydrogenase (GAPDH). Bars indicate the mean ± SEM and ^*∗*^ *p* < 0.05.

Data on histological findings in colons in patients with PSC and controls are summarized in Table 2. Ileal tissues were not assessed by the pathologist as they are not included in the scoring system applied in this study. Expression of* PXR* mRNA was similar in patients with PSC and controls regardless of the examined part of colon. The level of* SULT2A1* mRNA was significantly lower in the ileum of patients: 0.37 ± 0.1 in PSC without UC and 0.41 ± 0.1 in PSC with UC versus 1.01 ± 0.2 in controls,* p* = 0.02 and* p* = 0.03, respectively. This decrease was not seen in either ascending or sigmoid colon. These data are summarized in Figure 3.

### 3.3. Lack of the Alterations within Promoter Region of* SULT2A1* in PSC Patients

The genotyping analysis has shown that the examined SNPs, that is, rs11569683 and rs112433193, are not present among PSC patients. Furthermore, since earlier studies established that IR2 and DR4 motifs within promoter region of human* SULT2A1* are involved in the PXR-induced activity of the this gene [15], the detailed analysis of the nucleotide sequence of the promoter region of* SULT2A1* gene containing the PXR binding sites was carried out (Figure 1). The genomic analysis did not identify any changes within the examined region (data not shown).

### 3.4. Expression of miR-378a Is Considerably Enhanced in Livers of PSC Patients

A substantial increase in the level of microRNA miR-378a-5p in liver tissue of PSC patients (3,6-fold change;* p* = 0.0047 versus PBC) was seen. The observed changes were specific for PSC but not for PBC. The identified microRNA was predicted to target* SULT2A1* mRNA (http://mirdb.org/miRDB/).

## 4. Discussion

In the present study, we looked at the expression of pregnane-X-receptor (PXR) and sulphotransferase 2A1 (SULT2A1) in the livers of patients with PSC and PBC. We documented, for the first time,* in vivo* evidence of increased PXR expression in these conditions. We also found that, contrary to PBC, PXR activation is not accompanied by an enhanced expression of SULT2A1, suggesting a disease-specific impairment of SULT2A1 expression in PSC. Also, miRNA analysis suggested that SULT2A1 expression in PSC is likely regulated by miR-378a-5p, further indicating a pathogenic role for this miR in PSC.

Why PXR activation fails to boost SULT2A1 expression in PSC remains puzzling. PXR is a key member of the NRs family of ligand-modulated transcription factors. It binds (as a heterodimer with RXR) to response elements in the promoter region of target genes involved in stimulation of the bile acid detoxification machinery. Human PXR agonists include LCA, rifampicin, statins, and corticosteroids [16]. Although there is a wide evidence for NR interaction pathways in cholestatic conditions, most data derive from* in vitro* and animal studies in experimentally induced cholestasis. Data analyzing expression of NRs in patients with chronic cholestatic disorders are scarce.

*SULT2A1* is a target gene for PXR and plays an important role in PXR-mediated detoxification. Little is known about SULT expression in cholestatic liver diseases. A comprehensive analyses of the BA profile in serum from cholestatic patients has shown significant reduction of the amount of sulfated LCA present in patients with PSC [12] and reduced LCA sulfotransferase activity was detected in human livers with PSC [10, 17]. Such data are in line with the diminished potential of SULT2A1 expression following PXR activation noted in PSC. Our study showed that* PXR* mRNA and protein expression were significantly enhanced in PSC and PBC livers. To our knowledge,* in vivo* evidence of increased expression of* PXR* has been obtained for the first time. This finding is in agreement with data obtained in experimental cholestatic injury induced in animals, as well as* in vitro* observations. These cumulative data confirm that in the course of cholestatic processes the expression of BA-activated PXR protein increases in order to protect against BA toxicity. PXR was reported to be involved in pathogenesis of ICP [18, 19] and PSC [19] as well as in adaptation to cholestatic liver diseases, for example, in obstructive cholestasis [20]. With regard to PBC, limited data were obtained by Zollner et al., who have shown a repression of* CYP7A1* mRNA and elevation of MRP4 protein level in patients with PBC compared to controls. However, that study has found that expressions of NRs including PXR were not significantly changed [6].

Although, in response to cholestatic insult, PXR expression was significantly increased in both conditions, this increase was markedly less effective in our patients with PSC. The pathophysiological significance of this finding requires further investigation. PXR participates in diverse ways in transcriptional regulation of cytosolic SULTs. Treatment of human intestinal Caco-2 cells with activators of PXR leads to the induction of SULT2A1 in a hepatocyte nuclear factor 4- (HNF4-) dependent manner [15]. In a rodent model, stimulation of PXR expression reduced liver injury triggered by LCA administration in a SULT2A1-dependent manner [21]. Radominska et al. demonstrated that SULT2A1 is the only enzyme responsible for bile-acid sulphation in the human liver [21, 22]. In human hepatocytes the basal levels of SULT2A1 expression are relatively high [23, 24]. Thus, in normal conditions, bile acid sulphation is a very efficient elimination process in humans, and LCA, the most hydrophobic and toxic bile acid, is rapidly sulphated already on the first pass through the liver [25, 26]. Taking into account the biological importance of SULT2A1 in protecting against the dysregulation of homeostasis caused by cholestasis, we considered that it is worthy to study its expression. In our study, the overexpression of PXR was accompanied by elevation of SULT2A1 mRNA and protein levels in patients with PBC, but not in PSC. In the latter group of patients, mRNA and protein levels were unchanged in comparison to controls samples, though an induction of PXR proteins was observed in this group. This observation may suggest that, in PSC, the positive regulation pathway between PXR and SULT2A1 is disturbed, resulting in an impairment of sulphation capacity. This finding is in line with our previous observations demonstrating a reduction of sulphation capacity in PSC but not in PBC and controls [12]. LCMS/MS analysis of the concentrations of LCA and its SULT2A1 metabolite-LCA-S in patients with PSC, PBC and controls clearly demonstrated a decrease of the sulphation potential of LCA in PSC with a metabolic LCA-S/LCA ratio reduction noted in PSC when compared to both PBC patients and controls. LCA-S/LCA ratio was 5.01 in PSC as compared to 1.94 in PBC and 1.75 in controls [12].

The data on SULT2A1 expression in human cholestatic tissues are very scanty. Analysis of human livers from various chronic liver diseases such as PBC, PSC, autoimmune hepatitis, and alcoholic cirrhosis (but not in cryptogenic cirrhosis) has shown that SULT2A1 activity and concentration are significantly reduced when compared to normal livers. However, a detailed comparison among disease groups did not produce statistically significant results [10].

As already mentioned, the interplay between PXR and HNF4alpha has been shown to be of importance in regulation of* SULT2A1* gene expression [27]. Regardless of the enhanced expression of PXR, the transcript level of its target gene, that is,* SULT2A1*, was not changed in the livers of PSC patients. Therefore, we decided to perform the genomic analysis of* SULT2A1* promoter in order to find out whether the alteration in nucleotide sequence may be accountable for the lack of adequate level of this detoxification enzyme. We hypothesized that a possible change in DNA sequence within* SULT2A1* promoter region, more precisely in the proximity of HNF4alpha and PXR binding sites, could explain the lack of the increased expression of* SULT2A1* gene in PSC disease. However, our hypothesis proved to be wrong, as neither SNP genotyping assessments nor a detailed sequencing of* SULT2A1* promoter have provided evidence in support of our assumption.

Within the last decade, microRNA (miRNA) have emerged as a new class of small molecules that control intracellular gene expression at a posttranscriptional level. Increasing body of evidences confirms the fundamental role of miRNA in the physiological and pathological processes in the liver. Our study provide a new insight into SULT2A1-specific expression patterns that can be modulated by miR-378a-5p in PSC patients. The substantially increased expression of miR-378a-5p in PSC liver may be responsible for the observed lower level of SULT2A1 protein, as the identified microRNA was predicted to be involved in the regulation of* SULT2A1* gene expression. However further analysis is needed to understand the role of miR-378a-5p in cholestatic liver diseases like PSC.

In this study we have also analyzed expression of both* PXR* and* SULT2A1* mRNA in the intestine of patients with PSC. To our knowledge, expression of* PXR* in the intestinal tissue has not been analyzed so far in PSC. The tendency of colitis to primarily involve the caecum and right hemicolon in PSC patients is in contradistinction to non-PSC related ulcerative colitis in which the disease is always distal and supports the hypothesis that elements of the enterohepatic circulation are implicated in the pathogenesis of PSC-associated colitis [28]. We found a significantly decreased expression of* SULT2A1* mRNA but not* PXR* in the ileum of patients with PSC. This finding further supports the notion of impaired SULT2A1 function in PSC. On the other hand, we did not see any difference between analyzed groups in terms of expression of* PXR* and* SULT2A1* mRNA in the colon. As expected, patients with PSC with concomitant UC had significantly more pronounced inflammatory features on their histology; however, this did not appear to affect either* PXR* or* SULT2A1* mRNA. As already mentioned, there is no study in the literature dealing specifically with this issue. Thus, this finding requires further investigation and its interpretation is difficult at this point.

## 5. Conclusions

In conclusion, our results indicate disease-specificity of intrinsic PXR-coordinated hepatoprotective mechanism against BA toxicity. In contrast to PBC, PSC patients show an impaired signaling between PXR and SULT2A1. The observed increase in liver miR-378a-5p level, a negative posttranscriptional modulator of* SULT2A1* gene, could contribute to the pathogenic processes seen in this condition. Since most of the accessible research in this area was undertaken in a rodent model, which does not translate directly to humans, our data on PXR and SULT2A1 expressions in humans are novel and may have a future translational clinical repercussion. More research is needed to understand the enigmatic role of SULT2A1 in the development of liver disease [29, 30].

## Figures and Tables

**Table 1:** Demographic and laboratory features of patients with primary PBC and PSC included in the part of the study analyzing expression of PXR and SULT2A1 in explanted livers.

	Liver	
	PBC (*n* = 21)	PSC (*n* = 11)
Gender (M/F)	1/20	7/4
Age (mean ± SD. range)	56 ± 9 (36–69)	48 ± 14 (17–62)
AST (U/L)	148 ± 128	204 ± 127
ALP (U/L)	447 ± 296	541 ± 265
Bilirubin (*μ*mol/L)	114 ± 112	133 ± 102

**(a):** 

Features	Ascending Colon					
	Control	PSC	PSC + UC	PSC versus control	PSC + UC versus control	PSC versus PSC + UC
Architectural change	0.1 ± 0.1	0.4 ± 0.2	0.7 ± 0.2	NS	*p* = 0.015	NS
Chronic inflammatory infiltrate	0.4 ± 0.1	1.1 ± 0.2	1.5 ± 0.3	*p* = 0.01	*p* = 0.0003	NS
Lamina propria neutrophils and eosinophils	0.1 ± 0.1	0.5 ± 0.2	1.3 ± 0.3	NS	*p* = 0.001	*p* = 0.038
Neutrophils in epithelium	0.0	0.1 ± 0.1	0.3 ± 0.2	NS	NS	NS
Crypt destruction	0.0	0.0	0.5 ± 0.4	NS	NS	NS
Erosion or ulceration	0.0	0.4 ± 0.4	0.3 ± 0.3	NS	NS	NS
Total	**0.5 **±** 0.2**	**2.1 **±** 0.4**	**3.6 **±** 0.8**	***p*** =** 0.025**	***p*** <** 0.0001**	***p*** =** 0.038**

**(b):** 

Feature	Sigmoid colon					
	Control	PSC	PSC + UC	PSC versus control	PSC + UC versus control	PSC versus PSC + UC
Architectural change	0.1 ± 0.1	0.2 ± 0.2	0.9 ± 0.3	NS	*p* = 0.0015	*p* = 0.01
Chronic inflammatory infiltrate	0.4 ± 0.1	0.4 ± 0.2	1.3 ± 0.1	NS	*p* < 0.0001	*p* = 0.0003
Lamina propria neutrophils and eosinophils	0.0	0.1 ± 0.1	0.9 ± 0.2	NS	*p* < 0.0001	*p* = 0.0001
Neutrophils in epithelium	0.0	0.0	0.1 ± 0.1	NS	NS	NS
Crypt destruction	0.0	0.0	0.5 ± 0.4	NS	NS (*p* = 0.054)	NS
Erosion or ulceration	0.0	0.0	0.4 ± 0.3	NS	NS	NS
Total	**0.5 **±** 0.2**	**0.6 **±** 0.3**	**2.9 **±** 0.4**	**NS**	***p* < 0.0001**	***p* < 0.0001**

## List of Abbreviations

HNF4: Hepatocyte nuclear factor 4
LCA: Lithocholic acid
NR: Nuclear receptor
PBC: Primary biliary cirrhosis
PSC: Primary sclerosing cholangitis
PXR: Pregnane-X-receptor
SULT2A1: Sulphotransferase 2A1.
